# Biologic Sequencing in Systemic Lupus Erythematosus: After Secondary Non-response to Rituximab, Switching to Humanised Anti-CD20 Agent Is More Effective Than Belimumab

**DOI:** 10.3389/fmed.2020.00498

**Published:** 2020-08-27

**Authors:** Sabih Ul Hassan, Md Yuzaiful Md Yusof, Paul Emery, Shouvik Dass, Edward M. Vital

**Affiliations:** ^1^Leeds Institute of Rheumatic and Musculoskeletal Medicine, University of Leeds, Leeds, United Kingdom; ^2^NIHR Leeds Biomedical Research Centre, Leeds Teaching Hospitals NHS Trust, Leeds, United Kingdom

**Keywords:** B cells, belimumab, immunogenecity, rituximab, systemic lupus erythematosus

## Abstract

**Background:** Rituximab is commonly used for systemic lupus erythematosus (SLE) but secondary non-depletion and non-response (2NDNR) associated with anti-drug antibodies is a notable problem with repeat rituximab cycles. Other B cell-targeted therapies include other anti-CD20 monoclonal antibodies or belimumab.

**Objective:** To compare efficacy of switching to alternative anti-CD20 agents vs. belimumab in SLE patients with 2NDNR to rituximab.

**Methods:** One hundred and twenty five patients received rituximab and had evaluable data. 77/125 received repeat rituximab cycles. Of these, 14/77 (18%) had 2NDNR. 8/14 patients were switched to belimumab (CD20-to-belimumab group) and 6/14 patients were switched to an alternative humanised anti-CD20 agent (CD20-to-CD20 group, ocrelizumab *n* = 3, ofatumumab *n* = 2, obinutuzumab *n* = 1). Efficacy was assessed using the BILAG-2004, SLEDAI-2K, SRI-4, and daily prednisolone requirement at baseline and 6 months.

**Results:** In the CD20-to-belimumab group, only one patient achieved an SRI-4 and 2/8 patients had new/worsening BILAG-2004 grade A for lupus nephritis. There was no improvement in SLEDAI-2K; median (IQR) was 11.0 (9.5–14.8) at baseline and 10 (9.5–15.5) at 6 months. Median (IQR) prednisolone dose increased from 7.5 mg (4.4–12.5) to 10 mg (6.3–10). In the CD20-to-CD20 group, all 6 patients achieved an SRI-4. Median (IQR) SLEDAI-2K improved from 16.0 (10.3–24.0) at baseline to 5.0 (2.5–6.0) at 6 months. Median (IQR) prednisolone dose decreased from 15 mg (15–15) to 10.5 mg (5.3–15.0).

**Conclusion:** This is the first assessment of belimumab's efficacy in a post-rituximab population. Our data suggests that patients with 2NDNR to rituximab, which constituted 11% of all patients initiated on this drug, should be switched within the same biologic class to another anti-CD20 agent.

## Introduction

Rituximab, a chimeric monoclonal antibody (mAb) is commonly used off-label for the treatment of antibody positive systemic lupus erythematosus (SLE) in patients with severely active disease (including renal manifestations) despite conventional immunosuppressants such as mycophenolate mofetil and cyclophosphamide (1). Although two large phase III randomised controlled trials EXPLORER (non-renal) and LUNAR (renal) failed to meet their primary end points, rituximab appears to be effective in a large number of single-centre open label series (2–4), multi-centre registries (1, 5) and a systematic review of off-label use (6). We previously reported that clinical response to rituximab was better if complete B cell depletion, as measured using highly sensitive flow cytometry [HSFC (7)], was achieved (8). However, in patients with good initial response to a first cycle of rituximab, we found a substantial rate of secondary non-depletion and non-response (2NDNR). We previously defined this as a phenomenon whereby SLE patients who initially responded well to rituximab with B-cell depletion, subsequently experienced; a severe infusion reaction > 24 h during the second infusion of a cycle; failed to completely deplete B-cells; and did not clinically respond during repeat cycles. 2NDNR is associated with anti-rituximab antibodies. Since these patients often had severe disease resistant to other therapies, determining the best follow-on therapy in this situation is crucial.

It is logical to continue to target B cells in these patients given their prior good response to rituximab. There are two potential strategies. First, switching to an alternative anti-CD20 agent, particularly humanised (type I mAb: ocrelizumab, ofatumumab or type II mAb: obinutuzumab), has been reported with good clinical outcomes for the type I anti-CD20 mAbs (8–10) as well as *in vitro* for obinutuzumab (11). None of these anti-CD20 mAbs are currently licensed for use in SLE. Second, switching to belimumab as currently the only biologic agent licensed for treating SLE. Belimumab targets B cells indirectly via B cell activating factor (BAFF) inhibition. BAFF is not only a potent B cell activator, it also plays an important role in B cell proliferation and differentiation (12). Although it is licenced for treating antibody positive SLE with a high degree of disease activity (excluding active renal and neuro-psychiatric complications), its evidence for efficacy is mainly in biologic-naïve patients (13, 14). Neither option has previously been assessed in the context of 2NDNR to rituximab.

BAFF levels are known to significantly increase after B cell depletion, and this may assist in the survival of new B cells emigrating from bone marrow. BAFF levels have also been associated with relapse after rituximab (15). Based on these findings, several trials are in progress using a combination of rituximab and belimumab (16, 17). However, this treatment regimen and trial population are clearly distinct from the rituximab 2NDNR problem.

The objective of this study was to report the comparative efficacy of switching to either (i) belimumab, or (ii) alternative, humanised anti-CD20 agents in SLE patients with prior 2NDNR to rituximab. We hypothesised that both of these B cell targeted agents would have higher response rates in 2NDNR patients than for SLE patients without previous 2NDNR. However, our results showed a marked difference in their efficacy in this population.

## Methods

### Patients and Design

A prospective observational study was conducted of all patients with moderate to severe SLE [with at least 1 × British Isles Lupus Assessment Group (BILAG)-2004 grade A or 2 x BILAG-2004 grade Bs] who were treated with rituximab in Leeds between January 2004 and October 2019. Inclusion criteria were (1) age ≥ 18 years old; (2) fulfilling the revised 1997 American College of Rheumatology classification for SLE (18) and (3) at least 6 months follow-up post-rituximab and post-rituximab switch following a 2NDNR (defined below). Total follow up time on each therapy was calculated from the date of therapy initiation until the date of therapy discontinuation / death / last update of data in January 2020.

### Rituximab Therapy and 2NDNR

Rituximab (MabThera) was administered to patients if they had moderate to severe SLE despite prior therapy with either mycophenolate mofetil or cyclophosphamide, or with toxicity to these agents, in line with the NHS England criteria (19). Rituximab was administered as 2 × 1000 mg at weeks 0 and 2, each preceded by 100 mg methylprednisolone. Patients received repeat cycles of the same dose of rituximab if they had a clinical relapse, defined by at least 1 x new BILAG-2004 B, following an initial response at 6 months. In this cohort, we previously reported that 14% of patients with SLE who had previously depleted and responded well to rituximab, subsequently experienced (1) a severe infusion reaction > 24 h during the second infusion of a cycle, (2) failure to deplete CD20+ B cells (naïve and memory) and (3) clinical non-response during repeat cycles. We called this secondary non-depletion and non-response (2NDNR) (8). This phenomenon has also been reported by other groups (20).

### Rituximab to Belimumab Switch (CD20-to-Belimumab Group)

Treatment for 8 patients with 2NDNR to rituximab was switched to belimumab. Belimumab was administered using its licensed dose of 10 mg/kg at weeks 0, 2, 4 then every 4 weeks. It was discontinued in patients with non-response or if their condition worsened, requiring other therapies.

### Rituximab to Alternative Anti-CD20 mAb Switch (CD20-to-CD20 Group)

For 6 patients with 2NDNR, therapy was switched to a humanised anti-CD20 agent. This was chosen based on availability (compassionate supply from Roche, or individual funding from the NHS England). Three patients were treated with ocrelizumab 2 × 1000 mg at weeks 0 and 2, each preceded by 100 mg methylprednisolone; for 2 patients we treated with ofatumumab 2 × 700 mg at weeks 0 and 2, each preceded by 100 mg methylprednisolone; and 1 patient was treated with obinutuzumab 2 × 1000 mg at weeks 0 and 2, each preceded by 100 mg methylprednisolone.

Treatment choice was determined by availability and funding rather than clinical status. Patients were treated with alternative anti-CD20 agents before the NICE approval and when these agents were available. This depended on compassionate supply from manufacturers (ocrelizumab), individual funding applications until no longer available (ofatumumab), and funding by the hospital trust (obinutuzumab). From the date of the NICE approval for belimumab, patients received belimumab if they met NICE criteria.

### Clinical Outcomes

Data were collected as part of the Leeds Connective Tissue Disease and Vasculitis (CONVAS) observational study. Baseline characteristics including demographics, disease activity, previous and concomitant immunosuppressant and daily prednisolone use were collected. Treatment efficacy was assessed using the BILAG-2004 (21), SLE Disease Activity Index version 2000 [SLEDAI-2K (22)], and daily prednisolone requirement at baseline and 6 months after the follow-on therapy.

BILAG-2004 responses at 6 months were determined as follows: (1) major clinical response (MCR) = improvement of all domains rated A/B to grade C/better and no A/B flare between baseline and 6 months; (2) partial clinical response (PCR) = maximum of 1 domain with a persistent grade B with improvement in all other domains and no A or B flare; or (3) no clinical response (NCR) = those not meeting the criteria for major or partial clinical response. Global BILAG-2004 score was calculated as follows: grade A = 12, grade B = 8, grade C = 1, and grades D and E = 0 (23).

SLE Responder Index (SRI-4) was defined by a 4-point improvement in the SLEDAI-2K with no worsening in the BILAG-2004 or in the physicians' global assessment (24).

### Laboratory Assessments

Peripheral blood B-cell subsets (naïve, memory B-cells and plasmablasts) were measured using HSFC as previously described (7) at baseline and 6 weeks after treatment with rituximab or alternative anti-CD20 agents without knowledge of clinical status other than time since therapy. Complete B-cell depletion was defined as counts < 0.0001 × 10^9^/L and repopulation as ≥ 0.0001 × 10^9^/L. Anti-dsDNA titres and ENA profile (anti-Ro, -La, -Sm, -Scl-70, -Jo-1, -RNP, -Sm/RNP, -Ribosomal P, -Chromatin) were measured using ImmunoCAP™ chemiluminescent immunoassay by Thermo Fischer Scientific prior to July 2012 and Bioplex 2200 Immunoassay (after July 2012). Complement levels (C3 and C4) (normal range for C3: 0.75–1.65 g/L and for C4: 0.14–0.54 g/L) and total serum immunoglobulin titres were measured by nephelometry. All immunological tests above were analysed at an accredited NHS laboratory.

### Ethics Approval

This observational study was approved by the Leeds (East) Research Ethics Committee (REC), 10/H1306/88 and conducted in compliance with the Declaration of Helsinki. All patients gave written informed consent. The off-label use of rituximab, ofatumumab, ocrelizumab and obinutuzumab were all approved by the Leeds Teaching Hospitals NHS Trust Drug and Therapeutic Committee.

### Statistical Analyses

Descriptive statistics were summarised using median with interquartile range (IQR) for continuous variables and proportion for categorical variables. Continuous variables were compared using either Student's *t*-tests, Mann-Whitney's test or Kruskall-Wallis test depending on data type and distribution. All statistical analysis was performed using IBM SPSS Statistics v21.0 (IBM Corp, Armonk, New York, USA) and Graph Pad Prism V.6.01 for Windows.

## Results

### Demographics of Patients With 2NDNR to Rituximab

One hundred and twenty five patients with SLE received rituximab in Leeds over the 15 years follow-up and had evaluable data at 6 months. Of these, 100/125 (80%) had an initial BILAG-2004 response (MCR and PCR). 77/125 (62%) patients suffered a relapse and required repeat cycles of rituximab. Of these, 61/77 (79%) patients maintained BILAG-2004 response, 2/77 (3%) had secondary inefficacy, and 14/77 (18%) developed 2NDNR either in the second cycle (*n* = 10/77; 13%) or the third cycle (*n* = 3/40; 8%). Baseline characteristics of the 14 patients with 2NDNR to rituximab are summarised in Table 1. Patients who were switched to alternative anti-CD20 agents (CD20-to-CD20 group) were younger at the time of drug initiation, had shorter disease duration, lower number of previous oral immunosuppressants when compared to those who were switched to belimumab (CD20-to-belimumab group). However, the dose of concomitant oral prednisolone, median SLEDAI-2K scores, and the proportion of patients on concomitant anti-malarial and immunosuppressants (IS) were comparatively higher in the CD20-to-CD20 group.

**Table 1:** Baseline characteristics.

**Characteristic**	**CD20-to-Belimumab Group (*n =* 8)**	**CD20-to-CD20 Group (*n =* 6)**
Age (years), Median (IQR)	44.0 (31.5–56.8)	28.0 (23.3–35.0)
Female:Male	8:0	6:0
Ethnicity, n (%)		
Caucasian	5/8 (63)	1/6 (17)
Afro Caribbean	3/8 (37)	5/6 (83)
Disease duration at drug initiation (years), Median (IQR)	18.0 (12.8–20.0)	6.5 (6.0–8.5)
Previous Cyclophosphamide, n (%)	3/8 (37)	1/6 (17)
Number of previous oral immunosuppressants^*^, Median (IQR)	3.0 (2.5–5.0)	1.5 (1.0–2.8)
Prednisolone dose (mg), Median (IQR)	7.5 (4.4–12.5)	15.0 (15.0–15.0)
Concomitant antimalarial, n (%)	4/8 (50)	4/6 (67)
Concomitant IS^*^, n (%)	4/8 (50)	5/6 (83)
SLEDAI-2K, Median (IQR)	11.0 (9.5–14.8)	16.0 (10.3–24.0)
BILAG-2004 A/B^**^, n (%)		
General	1/8 (13)	3/6 (50)
Mucocutaneous	6/8 (75)	3/6 (50)
Neuropsychiatric	1/8 (13)	2/6 (33)
Musculoskeletal	6/8 (75)	3/6 (50)
Cardiorespiratory	1/8 (13)	0/6 (0)
Renal	1/8 (13)	4/6 (67)
Haematological	1/8 (13)	1/6 (17)

* *concomitant immunosuppressant (IS) = Azathioprine, Mepacrine, Methotrexate, Mycophenolate Mofetil or Tacrolimus*.

** *no patient had activity in gastroenterological or ophthalmic domains of BILAG-2004 so these data not shown*.

### Clinical Outcomes of the CD20-to-Belimumab Group

Eight patients received belimumab after rituximab. All were female with a median (IQR) age at the time of drug initiation of 44.0 years (31.5–56.8). Reasons for failure of rituximab in this subgroup were: (i) primary non-response (never responded) = 1/8; and (ii) 2NDNR = 7/8. At belimumab baseline, 6/8 patients had positive anti-dsDNA and low complement levels in line with data predicting better response to belimumab and the current UK National Institute for Health and Care Excellence (NICE) guidance (25, 26). The other two patients were treated prior to the publication of this guidance.

At 6 months post-belimumab, only one patient achieved an SRI-4. However, belimumab was discontinued for this patient at the 6-month time point due to recurrent chest and urinary tract infections. Another 2/8 patients had a 4-point reduction in SLEDAI-2K (22 → 18 and 14 → 10) but failed to achieve SRI-4 due to one having new BILAG-2004 activity in cardiorespiratory domain (Grade E → B) and worsening in general domain (Grade C → B), whilst the other had worsening of both mucocutaneous and renal domains (Grade B → A). A complete breakdown of BILAG-2004 domain scores at baseline and 6 months are shown in Table 2.

**Table 2:** BILAG-2004 scores.

**Baseline**										**6 months**								
**Patient**	**Biologic**	**Gen**	**Muco**	**Neuro**	**MSK**	**Cardio**	**Renal**	**Haem**	**Total score**	**Gen**	**Muco**	**Neuro**	**MSK**	**Cardio**	**Renal**	**Haem**	**Total score**	**BILAG-2004 Response**
**CD20-to-Belimumab Group**																		
1	Bel	E	A	E	B	E	E	C	21	E	B	E	B	E	E	C	17	PCR
2	Bel	E	B	E	E	E	E	C	9	E	B	E	E	E	E	C	9	NCR
3	Bel	A	C	E	C	D	E	B	22	D	C	E	C	E	A	B	22	NCR
4	Bel	D	B	E	B	D	B	C	25	D	A	E	D	D	A	C	25	NCR
5	Bel	D	B	E	B	D	E	C	17	D	B	E	B	D	E	C	17	NCR
6	Bel	C	B	D	A	A	E	D	33	C	C	D	B	D	E	D	10	PCR
7	Bel	E	E	B	A	D	E	C	21	E	E	B	A	D	E	C	21	NCR
8	Bel	C	B	E	A	E	E	C	22	B	B	E	C	B	E	C	26	NCR
**CD20-to-CD20 Group**																		
9	Ocr	B	A	E	B	D	A	C	41	D	C	E	D	D	D	C	2	MCR
10	Ocr	B	E	E	D	E	A	C	21	D	E	E	D	E	D	C	1	MCR
11	Ocr	E	E	B	B	E	E	C	17	E	E	D	C	E	E	C	2	MCR
12	Ofa	E	E	E	E	E	A	A	24	E	E	E	E	E	B	C	9	PCR
13	Ofa	B	A	B	B	E	A	C	49	D	C	D	D	E	C	C	3	MCR
14	Obi	D	A	D	C	E	D	C	14	D	C	D	C	E	D	C	3	MCR

*Ocr, Ocrelizumab; Ofa, Ofatumumab; Obi, Obinutuzumab; MCR, major clinical response; PCR, partial clinical response; NCR, no clinical response*.

**Figure 1:**
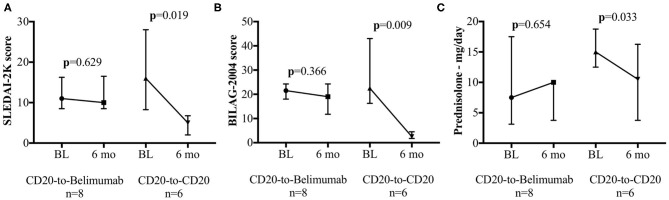
SLEDAI-2K, Global BILAG-2004, and Daily prednisolone dose. Clinical efficacy assessments for the CD20-to-belimumab and the CD20-to-CD20 groups. Each figure shows the pre- and post-treatment results for the **(A)** SLEDAI-2K, **(B)** total BILAG score, and **(C)** daily oral prednisolone requirements in the CD20-to-CD20 group compared to the CD20-to-belimumab group. Points represent median and error bars denote interquartile range. BL, Baseline; 6 mo, 6 months.

There was no significant improvement in the SLEDAI-2K post-belimumab; median (IQR) at baseline and 6 months were 11.0 (9.5–14.8) and 10 (9.5–15.5), respectively; *p* = 0.629 (Figure 1A). There was no improvement in the Global BILAG-2004 score post-belimumab; median (IQR) at baseline and 6 months were 21.5 (20.0–22.8) and 19.0 (15.3–22.8), respectively; *p* = 0.366 (Figure 1B). Median (IQR) prednisolone dose had increased from 7.5 mg (4.4–12.5) at baseline to 10 mg (6.3–10) at 6 months; *p* = 0.654 (Figure 1C).

Notably, there were two new episodes of lupus nephritis during belimumab therapy (1 = relapse with Class III nephritis and 1 = *de novo* Class II and V nephritis). Treatment for both patients was switched to intravenous cyclophosphamide therapy.

Total follow up time on therapy in this group was 9.9 patient-years. 4/8 patients continued therapy for longer than 6 months and they all received increased doses of immunosuppressant and prednisolone. Of these, 1/4 patient had initial BILAG-2004 PCR but developed flare of lupus nephritis and stopped at 11 months; 1/4 patient did not meet either an SRI-4 or BILAG-2004 response at 6 months but had BILAG-2004 PCR at 9 months and discontinued therapy at 24 months due to secondary inefficacy; 1/4 patient had two lengthy interruptions to therapy due to unrelated surgical procedures leading to cessation of belimumab at 24 months; and 1/4 patient remains on belimumab at 3 years but still not in clinical remission despite requiring escalation of concomitant oral immunosuppressants and prednisolone.

### Immunological Outcomes of the CD20-to-Belimumab Group

**Figure 2:**
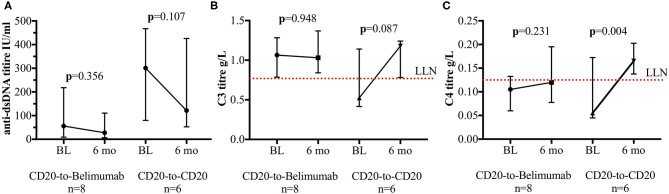
Anti-dsDNA titres and complement levels. Immunological tests for the CD20-to-belimumab and the CD20-to-CD20 groups. Each figure shows the pre- and post-treatment for the **(A)** anti-dsDNA titres; **(B)** complement C3 level and **(C)** complement C4 level. Points represent median and error bars denote interquartile range. The dotted red lines represent lower limit of the normal values of the tests. BL, Baseline; 6 mo, 6 months.

Of 6/8 patients with increased anti-dsDNA titre at belimumab baseline, none achieved normalisation of anti-dsDNA titre at 6 months. Furthermore, anti-dsDNA titre did not significantly improve post-belimumab; median (IQR) at baseline and at 6 months were 56 IU/mL (21–132) and 27.5 IU/mL (14.8–104.3), respectively; *p* = 0.356 (Figure 2A).

For complement C3 level, median (IQR) at baseline and at 6 months were 1.07 g/L (0.84–1.17) and 1.03 g/L (0.88–1.21), respectively; *p* = 0.948 (Figure 2B). 1/8 patient with low level at baseline did not improve post-belimumab. For complement C4 level, median (IQR) at baseline and at 6 months were 0.11 g/L (0.08–0.12) and 0.12 g/L (0.09–0.17), respectively; *p* = 0.231 (Figure 2C). 6/8 patients had low level at baseline. Of these, only 1/6 had normalisation of C4 level at 6 months post-belimumab. C3 and/or C4 levels which were normal at baseline (*n* = 2), remained within normal range at 6 months post-belimumab.

### Clinical Outcomes of the CD20-to-CD20 Group

Following 2NDNR to rituximab, treatment for 6 patients was switched to humanised anti-CD20 mAbs (3 = ocrelizumab, 2 = ofatumumab, and 1 = obinutuzumab). All 6 patients were female with a median (IQR) age at the time of drug initiation of 28.0 years (23.3–35.0).

Total follow up time on therapy in this group was 31.8 patient-years. Six weeks after treatment with the alternative anti-CD20 agent, complete B cell depletion was achieved in 5/6 patients, while the remaining one had substantially reduced total B cell counts (0.0016 × 10^9^/L).

At 6 months post-switch to alternative anti-CD20 agents, all patients achieved an SRI-4. The median (IQR) SLEDAI-2K score had improved from 16.0 (10.3–24.0) at baseline to 5.0 (2.5–6.0) at 6 months; *p* = 0.019 (Figure 1A). The Global BILAG-2004 score significantly improved from 22.5 (18.0–36.8) at baseline to 2.5 (2.0–3.0) at 6 months; *p* = 0.009 (Figure 1B). This is also reflected in the BILAG-2004 response, which was MCR for 5/6 patients and PCR for the remainder 1/6 patient, vs. no MCR in the CD20-to-Belimumab group. In fact, 3/8 patients in the CD20-to-Belimumab group had a worsening of BILAG-2004 response. A complete breakdown of BILAG-2004 domains at baseline and 6 months post-alternative anti-CD20 mAbs switch are shown in Table 2. Furthermore, median (IQR) prednisolone dose had decreased from 15 mg (15–15) at baseline to 10.5 mg (5.3–15.0) at 6 months; *p* = 0.033 (Figure 1C).

### Immunological Outcomes of the CD20-to-CD20 Group

In all 6 patients, the anti-dsDNA titres had reduced at 6 months, however none normalised; median (IQR) at baseline and at 6 months were 301.0 IU/ml (137.5–342.3) and 121.5 IU/ml (74.8–259.0), respectively; *p* = 0.107 (Figure 2A). For complement C3 and C4 levels, 4/6 had low levels for both at baseline. Of these, 3/4 had normalisation of the C3 and C4 levels at 6 months (Figures 2B,C). Those C3 or C4 levels which were normal at baseline (*n* = 2), remained within normal range at 6 months post-alternative anti-CD20 mAbs switch. Median (IQR) for complement C3 level at baseline and at 6 months were 0.53 g/L (0.45–0.89) and 1.18 g/L (0.91–1.21), respectively; *p* = 0.087. For complement C4 level at baseline and at 6 months, median (IQR) were 0.06 g/L (0.05–0.13) and 0.17 g/L (0.15–0.18), respectively; *p* = 0.004.

## Discussion

In this study, we report the first evidence on biologic switching in SLE, suggesting an important difference in response after rituximab. These data highlight the importance of appropriate biologic sequencing in this growing resistant subgroup.

Many potential therapeutic targets have been explored for SLE. The most prominent target the B cell pathway directly. Most pharmacological agents targeting B cells either deplete them (rituximab, ocrelizumab, ofatumumab, and obinutuzumab); or inhibit BAFF (belimumab, tabalumab, atacicept). Non-B cell targets are diverse and include type I interferon (IFN-I) (27), interleukin (IL) 12/23, CTLA4-CD28 co-stimulation and the Janus kinase/signal transducers and activators of transcription (JAK-STAT) pathway. These agents may still impact on B cells, but only indirectly.

The diversity of these potential targets raises the question of the most appropriate follow-on therapy in patients who either fail to respond to their first biologic or, lose an initial good response (2NDNR). The answer to this question depends on our understanding of the mechanism for inadequate response.

For most manifestations of SLE, we showed that a key determinant of rituximab response is the degree of B cell depletion achieved with therapy (8). This implies that these clinical manifestations are B cell-dependent. Some manifestations of SLE appear to be non-B cell mediated. We showed that most discoid lupus erythematosus and some subacute cutaneous lupus erythematosus lesions either did not respond, worsened or initiated during rituximab therapy despite complete peripheral B cell depletion (28).

Belimumab may also be more effective in a subgroup of SLE patients in whom B cells have a more dominant role. In randomised clinical trials, the difference in response between belimumab and placebo is twice as large in patients with B cell biomarkers (i.e., raised anti-dsDNA titres and low complement levels) (29).

Thus, for patients initially responding well to rituximab (i.e., have proven B cell-dependent manifestations) but subsequently develop pharmacodynamic resistance (2NDNR), switching to any other B cell targeted therapy would appear to be an appropriate strategy. We therefore expected a *higher* SRI-4 response rate than the ~55% of unselected SLE patients who responded in the pivotal trials of belimumab (12, 13, 30) given the resultant raised BAFF levels following successful depletion of B-cells. We also expected a high response rate to an alternative B cell depleting therapy, provided that depletion could be adequately restored. Surprisingly, we found that the SRI-4 response rate to belimumab was markedly worse in our study than in its trials, especially noting the new episodes of lupus nephritis.

These results may reveal potential complexities of therapeutic BAFF inhibition. After rituximab, there is a marked elevation of serum BAFF; up to 10-fold normal levels (31). Simultaneously, there is a marked shift in B cell dynamics with sustained reduction of naïve B cells that bear the BAFF-R receptor. Thus, there are relatively greater proportions of memory B cells and plasmablasts that express tumour necrosis factor receptor superfamily member 13b (TACI) and B cell maturation antigen (BCMA) receptors instead. These memory B cells and plasmablasts also bind APRIL (3). In particular, TACI signalling may have more complex effects on B cells than the BAFF-R signalling that predominates in rituximab-naïve SLE patients (32). Given these changes, it may therefore be expected that the effects of BAFF blockade in this situation might differ from the general SLE population in which the drug was evaluated in phase III trials.

In contrast, the effectiveness of alternative anti-CD20 agents in patients with 2NDNR to rituximab is entirely consistent with our hypothesis and the correlations we have reported between the degree of B cell depletion and clinical response. Anti-drug antibodies are more likely to occur against chimaeric mAbs. Regular cycles of rituximab may theoretically prevent their development, but the long treatment intervals required for some SLE patients, as well as the underlying propensity for B cell activity and antibody formation in SLE may account for their higher frequency in SLE compared to other diseases in which rituximab is used. The alternative anti-CD20 agents used in this study were humanised or fully human. There may be other differences between anti-CD20 agents that affect the efficacy of depletion in SLE, which may be particularly important for obinutuzumab (33).

These results may not affect the use of belimumab in SLE patients in general, nor the rationale for the various rituximab-belimumab combination strategies in clinical trials (34). Neither do they provide a comparison of the efficacy of these agents in their more typical patient populations. However, this study emphasises the importance of gathering data on belimumab in real world settings and deeper understanding of the BAFF pathway in SLE.

This study has some limitations. First, the sample size was small to definitively confirm the efficacy of the two strategies used in patients with 2NDNR to rituximab. These findings need to be validated in larger patient cohorts. However, the size of the difference seems large since only one patient in the CD20-to-belimumab group achieved the SRI-4 compared to all of the patients treated with alternative anti-CD20 agents. Second, this study was non-randomised. There was an imbalance in some of the baseline clinical characteristics, particularly patients in the CD20-to-CD20 group who had worse SLE (i.e., higher SLEDAI-2K and higher concomitant daily prednisolone). However, this group still showed better response to therapy compared with the CD20-to-belimumab group. There were some patients with renal involvement at baseline in the CD20-to-CD20 group and not the belimumab group. However, since we observed new episodes of nephritis in the CD20-to-belimumab group, it does not seem promising to further investigate this line of therapy in more severe patients. Our patients were all recruited from the same population and assessed in the same way in a single centre with marked differences in response rates. Lastly, concomitant therapy with immunosuppressant were used in 83% of the patients, thus the overall efficacy could not be attributed to the alternative anti-CD20 agents alone. Nevertheless, our long-term follow-up of a large cohort of rituximab-treated patients is one of the best sources of data available for these more complex questions. It is unlikely that randomised trials will ever be completed for this question. In the absence of randomised trials, and with a clinically important problem, it is appropriate to use the best case series evidence available.

In conclusion, 2NDNR is an increasingly common problem in SLE patients treated with rituximab, often with severe disease. For these patients, our data suggest biologic therapy should be switched within the same class; i.e., to another anti-CD20 agent. This study demonstrates the importance of stratification of therapy in SLE, based on an understanding of the determinants of response.

## Data Availability Statement

All datasets presented in this study are included in the article/supplementary material.

## Ethics Statement

The studies involving human participants were reviewed and approved by Leeds (East) Research Ethics Committee (REC), 10/H1306/88. The patients/participants provided their written informed consent to participate in this study.

## Author Contributions

SH, MM, EV, PE, and SD: substantial contributions to the conception or design of the work, or the acquisition, analysis or interpretation of data, drafting the work or revising it critically for important intellectual content, and final approval of the version published. SH, MM, and EV: agreement to be accountable for all aspects of the work in ensuring that questions related to the accuracy or integrity of any part of the work are appropriately investigated and resolved. All authors contributed to the article and approved the submitted version.

## Conflict of Interest

SD has received honoraria from Roche and GSK. PE has received consultant fees from BMS, Abbott, Pfizer, MSD, Novartis, Roche, and UCB. He has received research grants paid to his employer from Abbott, BMS, Pfizer, MSD, and Roche. EV has honoraria and research grant support from Roche, GSK, and AstraZeneca. The remaining authors declare that the research was conducted in the absence of any commercial or financial relationships that could be construed as a potential conflict of interest.
